# The Questionnaire D-RECT German: Adaptation and testtheoretical properties of an instrument for Evaluation of the learning climate in medical specialist training

**DOI:** 10.3205/zma000997

**Published:** 2015-11-16

**Authors:** Peter Iblher, M. Zupanic, T. Ostermann

**Affiliations:** 1 University of Lübeck, Clinic for Anaesthesiology and Intensive Care Medicine, Lübeck, Germany; 2 Witten/Herdecke University, Institute for Teaching and Educational Research in Health Sciences, Witten, Germany; 3 Witten/Herdecke University, School of Medicine, Student Dean’s Office, Witten, Germany; 4 Witten/Herdecke University, Department of Psychology, Chair for Statistics and doctrine of methodology, Witten, Germany; 5 Witten/Herdecke University, Institute for Integrative Medicine, Witten, Germany

**Keywords:** D-RECT, learning climate, further medical education, residents, validation, replication study

## Abstract

**Aim: **Boor et al [1] developed and validated the questionnaire D-RECT (Dutch Residency Educational Climate Test ) to measure the clinical learning environment within the medical specialist training. In this study, a German version of this questionnaire (D-RECT German) is analyzed regarding testtheoretical properties.

**Problem: **Are the results of Boor et al replicable as a proof for validity of the questionnaire D-RECT?

**Material & Methods: **The study was performed as online survey using the questionnaire D-RECT German (50 items in 11 subscales). To determine item characteristics and internal consistency (Cronbach’s α), item- and reliability analyses were performed. Furthermore, a confirmatory factor analysis was performed using a model for maximum-likelihood estimation to evaluate validity.

**Results: **This replication study on the psychometric properties of the D-RECT with 255 residents at 17 German hospitals revealed heterogeneous discriminatory power for all items and an internal consistency of Cronbach’s α between 0.57 and 0.85. Within the confirmatory factor analysis, 6 items showed standardized regression coeffizients <0.5, two of them in the subscale “Attendings role”. Furthermore, strong interdependencies (>0.7) were found between the subscales “Supervision”, “Coaching” and “Attendings role”.

**Conclusion: **The present replication study with the D-RECT German showed structural differences with respect to factorial validity underpinning the need of further validation studies.

## Zusammenfassung

**Zielsetzung: **In der Arbeitsgruppe von Boor et al. [1] wurde der Fragebogen D-RECT (Dutch Residency Educational Climate Test) zur Erfassung des Lernklimas in der ärztlichen Weiterbildung entwickelt und validiert. Die deutschsprachige Version (D-RECT-German) wird in der vorliegenden Studie testtheoretisch überprüft.

**Fragestellung: **Ist eine Replikation der Ergebnisse aus der Originalarbeit von Boor et al. als Beleg der Validität des D-RECT möglich?

**Methodik: **Die Befragung erfolgte onlinebasiert mit dem Fragebogen D-RECT-German. Mit Item- und Reliabilitätsanalysen wurden die Kennwerte der 50 Items in 11 Subskalen sowie die interne Konsistenz (Cronbach’s α) ermittelt. Die Validitätsprüfung erfolgte mit einer konfirmatorischen Faktorenanalyse unter Verwendung eines Maximum likelihood basierten Strukturgleichungsmodells.

**Ergebnisse:** Die Itemanalysen dieser Replikationsstudie mit 255 WBA an 17 deutschen Krankenhäusern ergaben für die Items heterogene Trennschärfen, auch die interne Konsistenz der Subskalen zeigte variable Werte für Cronbach’s α zwischen 0.57 und 0.85. In der konfirmatorischen Faktorenanalyse wiesen 6 Items standardisierte Regressionskoeffizienten <0.5 auf die vorgegebenen Dimensionen auf, von denen zwei im Konstrukt „Einstellung der betreuenden Fachärzte“ zu finden waren. Die Korrelationen der Faktoren untereinander wies mit Korrelationen von über 0.7 starke Interdependenzen zwischen den Faktoren „Supervision“, „Coaching“ und „Einstellung der betreuenden Fachärzte“ auf.

**Schlussfolgerungen:** In der Replikationsstudie mit dem D-RECT-German für den deutschsprachigen Raum zeigten sich strukturelle Unterschiede hinsichtlich der faktoriellen Validität, so dass weitere Validierungsstudien für den internationalen Vergleich notwendig sind.

## 1. Background

Studying medicine has been subject to a considerable change within the last years. Clear and decisive efforts have been made to improve education of young physicians by means of medical didactics, leading to a changing perception of the priority of excellent teaching. This manifests in a variety of funding activities [2]. 

The license to practice medicine (approbation) as completion of this phase of education forms the initial basis for further qualification of the students with the task to enhance their skills and knowledge; and consequently to receive a specialist certification. In this context the demands regarding clinical competence and learning performance, which as a matter of course are consented as an imperative for medical education of students [3], [4], should also be applied for further medical education. Thus a sound and comprehensive specialist training has been explicitly expressed by the German Medical Association (http://www.bundesaerztekammer.de/fileadmin/user_upload/downloads/20130628-MWBO_V6.pdf, accessed 21.10.2015) and should be scrutinized, to enable the educators to interpret their status quo and to consequently put modifications into practice and reassess them again. A central basis for this approach is given by the various forms of learning atmosphere [5]. Of note it would be desirable to assess the impact of learning climate by means of a flexible, established and reliable instrument, which maps the relevant dimensions of the learning atmosphere, unfolds strengths and weaknesses of further education concepts and which also could be applied within educational research.

### The D-RECT questionnaire to evaluate residents’ learning climate

Based on qualitative studies on the establishment of an optimal learning climate in further medical education the working group of Boor et al. found the following three interacting domains:

1. Working environment,
2. Further education,
3. Needs of residents.

Within this theoretical construct an instrument to measure the clinical learning environment was developed which contains eleven categories (Dutch Residency Educational Climate Test/ D-RECT) [1] (see also attachment 1 ). The authors concluded further investigations with respect to the validity of the questionnaire for the use in international settings would be necessary. Up to now the factorial validity of the final questionnaire still remains to be analyzed. The original questionnaire D-RECT was translated by Boor et al into English for publication reasons.

Thus, our replication study investigated the application of the D-RECT in the German-speaking area, framing the following questions:

1. Examination of the test-theoretical properties of a German Version of the D-RECT (D-RECT-German) to investigate learning climate by means of item- and reliability analyses to derive its internal consistency (Cronbach’s α).
2. Investigation of factorial validity of the D-RECT-German by means of confirmatory factor analysis.

## 2. Material and Methods

Our study received a positive vote from the ethics-committee of the University of Witten/Herdecke. All participants were informed prior to the study and were given the opportunity to withdraw from the study. Agreement of participation was given on the basis of conclusive conduct. 

### 2.1. The D-RECT questionnaire

The D-RECT consists of 50 items in 11 subscales (see attachment 1 ) using a five-point Likert scale (1=does not apply – 5= totally applies). The English version of the D-RECT was translated by a bilingual native speaker from English to German and back again. In addition to the original items, information on age, gender, specialist area, hospital and training year were inquired. The study was performed as online survey using Lime Survey. All collected data was anonymized before statistical analysis. 

#### 2.2. Test theoretical validation of the D-RECT-German

To determine item characteristics, item means (M), standard deviation (SD) and discrimination (r_it_) were calculated. Discrimination is calculated as the correlation between the question score and the overall assessment (item-total-correlation) for each of the 11 subscales [6]. Item-total correlations between 0.4 and 0.7 are considered as good, between 0.2-0.4 as acceptable, between 0.1-0.2 as marginal and between 0 and 0.1 as unsatisfactory [7]. To determine scale characteristics and scale intercorrelations, scale means (M), standard deviation (SD), coefficients of homogeneity (Cronbach’s α) and corrected inter-scale-correlations (r according to Pearson) were calculated. For group comparisons a Cronbach’s α of 0.7 can be regarded as satisfactory; a value of Cronbach’s α greater than 0.8 as good [8]. In addition, Kaiser-Meyer-Olkin (KMO) measure of sampling adequacy and Bartlett’s test for sphericity were calculated to determine whether a confirmatory factor analysis was warranted.

Finally structural equation modeling was applied to determine, the amount of interdependency between items and constructs using the existing factorial solution as a model for maximum-likelihood estimation. The Chi-Square value served as a parameter for model validity. A significant Chi²-Test indicates a poor model fit. In addition common incremental measures of scale fit in structural, equation modeling like the Comparative Fit Index (CFI), Tucker Lewis Index (TLI) und Root Mean Square Error of Approximation (RMSEA) were calculated. For CFI and TLI values greater than 0.90 point to a good model-fit, while, a RMSEA>0.08 indicates a high amount of unexplained variance. Associations between items and given dimensions were expressed using standardized regression coefficients r_s_, which in case of r_s_<0.5 were judged as unsatisfactory. Correlations between the dimensions were determined by correlation coefficients from the estimated covariance matrix. Correlations>0.7 pointed towards an interdependency of the factors. Using the R-procedure *modindices,* possible source of misspecification of the model were identified. Due to the low sample size no further multi-level analyses were carried out. All calculations were run with SPSS 22, AMOS 20 and R.

## 3. Results

Our sample included 255 residents (female: n=129/50.6%; male: n=126/49.4%) of 17 German hospitals (see Table 1 (Tab. 1)) and from four medical areas (see Table 2 (Tab. 2)) with a mean age of 32±6 years. Differentiation with respect to years of medical training is given in table 3 (Tab. 3).

Results of item analysis are provided in attachment 1 . High agreements with mean values higher than 4.0 were given in Item 3 (“It is clear which attending supervises me.”; M: 4.3±0.9), Item 35 (“When I need a attending, I can always contact one.”; M: 4.4±0.8) and Item 36 (“When I need to consult a attending, they are readily available.”; M: 4.2±0.8). Lowest agreement was given for Item 10 (“My attendings occasionally observe me taking a history.”; M: 1.4±0.8), Item 13 (“Observation forms (i. e. Mini-CEX) are used to structure feedback.”; M: 1.1±0.6), Item 14 (“Observation forms (i. e. Mini-CEX) are used periodically to monitor my progress.”; M: 1.1±0.6), Item 44 (“In this rotation evaluations are useful discussions about my performance.”; M: 1.6±1.4), Item 45 (“My plans for the future are part of the discussion.”; M: 1.7±1.5) and Item 46 (“During evaluations, input from several attendings is considered.”; M: 1.3±1.3) with mean values lower than 2.0. 

All items showed a satisfying discriminatory power with none of the items being below the critical value of 0.2 (see attachment 1 ). 

Results of the scale analysis found high agreement of the residents with the subscale “Patient sign out” (mean±SD: 4.1±0.9). Lowest congruence was given for the scales “Feedback” (mean±SD: 1.5±0.5) and “Role of the specialty tutor” (mean±SD: 1.7±1.1). With respect to internal consistency, all subscales only showed a critical to moderate values of Cronbach’s α between 0.57 und 0.85. 

Examination of factorial structure of the D-RECT using confirmatory factor analysis resulted in an unsatisfactory model-fit with a highly significant chi-square value of 2383,576 (p<0.001). Moreover incremental-fit parameters CFI (0.768) and TLI (0.746) by no means reached the area of a good approximative model-fit. Only the absolute model-fit RMSEA of 0.068 revealed a sufficient matching of the postulated factorial structure with the empirical data. 

Correlations of the items and the preset dimensions of the structural equation model resulted in values between 0.225 and 0.957 implying a considerable heterogeneity. Six of the items showed standardised regression coefficients lower than 0.5 with the preset dimensions, of which two were found for the subscale “Attendings’ role”. Sufficient factor loadings were given for the dimensions “Supervision” (*r_s_* between 0.570 and 0.720), “Teamwork” (*r_s_* between 0.598 and 0.716), “Professional relations between attendings” (*r_s_* between 0.596 and 0.700), “Formal education” (*r_s_* between 0.557 and 0.842), “Role of the specialty tutor” (*r_s_* between 0.531 and 0.817) and “Patient sign out” (*r_s_* between 0.596 and 0.780). One loading below 0.5 was found in the dimensions “Coaching and assessment” (CA7: *r_s_*=0.416), “Feedback” (FB1: *r_s_*=0.225), “Peer collaboration” (PC3: *r_s_*=0.441), and “Work is adapted to residents’ competence” (WA3: *r_s_*=0.294). Two loadings below 0.5 were found for the dimension “Attendings’ role” (AR3: *r_s_*=0.470 and AR8: *r_s_*=0.359) (see Table 4 (Tab. 4)). This result is also confirmed by the analysis of misspecifications of the model. Here once again the item FB1 parallely loads on seven different dimensions and has to be considered as critical for the German Version of the D-RECT. 

Correlation analysis between the dimensions with one exception (“Teamwork” vs. “Role of the specialty tutor” r=-0.025) found positive correlations between the scales. In particular the subscale “Coaching and assessment” revealed the highest correlations with “Attendings’ role” (r=0.788) and “Feedback” (r=0.752). But also the scales “Attendings’ role” and “Work is adapted to residents’ competence” showed a critically high correlation of r=0.602. All other scales correlated in an acceptable discriminatory range below 0.6. Subscale 11 (“Attendings’ role”) exhibited the highest correlations with the other subscales (see Table 5 (Tab. 5)).

## 4. Discussion

This replication study on the psychometric properties of the D-RECT revealed acceptable to good discriminatory power for all items and an internal consistency of Cronbach’s α between 0.57 and 0.85. However, confirmatory factor analysis uncovered significant weaknesses in the construct. This suggests that the underlying model only fitts unsatisfactory with the empirical data. The following discussion is based on these results. 

Compared to the initial study sample of Boor et al., the present replication study recruited less residents (255 residents vs. 600 residents). Nevertheless, sample size exceeded the lower bound of five residents per Item and therefore was regarded as big enough for a factor analysis. In this respect, the replication study has presuppositions comparable to the study of Boor et al. [1]. While the sample of Boor et al. recruited its participants out of 26 specialist areas, residents of the replication study were recruited from the field of anesthesiology, internal medicine, pediatric surgery and family medicine (see Table 2 (Tab. 2)). With respect to item response, only subscale 4. “Teamwork” had comparable distributions, while almost all other scales had lower item values in the replication study (see attachment ). For the subscale 3 “Feedback” (items 13 & 14) this may be explained by the fact, that the included hospitals had not established standardized surveillance sheets (i.E. Mini-CEX). 

Apart from this scale, low values (<2) were, yet found in item 10 (“My attendings occasionally observe me taking a history.”) and in almost all items of subscale 10 “Role of the specialty tutor” (items 42-46). This contrasts the results of Boor et al. who did not find values below 3.0 in the respective items (except for item 10). The study of van Vendeloo et al. framed in the setting orthopedic further education, they also found a global mean of the D-RECT of 3.8±0.4 [9]. In general the dutch samples assessed the global learning climate of further medical education higher than our sample. However, it cannot be ruled out that this mean difference might be due to a specific job-related effect. Thus, it has to be questioned whether specialist training in different disciplines are comparable in principle. Another interpretation simply takes into consideration that such offers are of poorer quality in Germany. Therefore, apart from a possible selection bias a county specific impact on medical training has to be taken into account.

Regarding the internal consistency as a parameter for the reliability of the instrument, the current study shows lower values in five of the subscales, similar values in two subscales and higher values in four subscales compared to the original work of Boor et al. Apart from the subscales “Supervision”, “Feedback”, “Peer collaboration” and “Work is adapted to residents’ competence”, which lay below a Cronbach‘s alpha of 0.6, all other subscales in our study had a sufficiently high Cronbach’s α of at least 0.7 [8]. In particular all subscales with six to eight items showed good coefficients above a value of 0.8, whereas all other subscales failed to pass the lower bound of 0.7 [6]. 

Discriminatory power in all items was above 0.3. Thus it can be concluded that the D-RECT can distinguish between participants with low (unfavourable appreciation of learning climate) and high scores (good appreciation of learning climate). 

Confirmatory factor analysis uncovered fundamental weaknesses in the eleven-factorial model of Boor et al. Analysis of loading weight as well as intercorrelation of the subscales point towards a different underlying structure of the D-RECT-German compared with the original instrument. In particular the scales “Supervision”, “Attendings’ role” and “Coaching and assessment” showed a high amount of interdependency. With respect to item loadings the scales “Attendings’ role” and “Coaching and assessment” should be critically scrutinized on the basis of our results. A not reported explorative factor analysis with oblique rotation supported the inconsistency within the factors. This first of all has to be interpreted that there is insufficient evidence for validity of the international version of the D-RECT questionnaire. Therefore, the use of the D-RECT in the German speaking area should only be considered after stable replication of the results in further studies. In the light of the available evidence an international comparability, as discussed by Boor et al., is not given. Further validation studies with the original questionnaire are absolutely necessary for D-RECT to meet the requirements for a valid evaluation instrument. As a restriction for international comparisons, only the global score of the questionnaire can be used while the interpretation on the level of subscales is only partially supported by the current evaluation. 

Subsequent Dutch studies evaluated external validity of the D-RECT by means of correlation of the D-RECT with other scales: an actual study of Lombarts et al. found correlations between the global score of the D-RECT and the quality of teaching questionnaire SETQ (modified SFDP26-questionnaire) [10]. Apart from significant positive correlations of the mean scores of both scales, quality of teaching (SETQ) also correlated with the D-RECT subscales 2. “Coaching and assessment”, 7. “Work is adapted to residents’ competence” and 9. “Formal education”. In addition the study of van Vendeloo et al. found, that high global scores of the D-RECT were associated with better quality of life, higher work-life balance, less symptoms of emotional exhaustion and less signs of depersonalisation [9]. Although these results are interesting, they are not sufficiently proved at last. After clarification of the validity of the D-RECT they should be taken into consideration for further research in the field of medical education.

### Limitation

Although the current study fulfills the criteria of study quality as provided in Boor et al., the diverging number of specialist areas involved in the studies has to be taken into account. More focused future research with respect to the individual disciplines might safely rule out subject specific differences. Due to the low sample size appropriate multilevel models were not applied in the present study. In both studies a selection-bias cannot be excluded due to voluntary participation of the residents. Finally country specific differences in further education should be taken into consideration, which in the present study design could not be differentiated due to methodological issues. 

## 5. Conclusion

The present study investigated the psychometric properties of the German replication of the D-RECT questionnaire by means of reliability analyses and confirmatory factor analysis. We found structural differences with respect to factorial validity underpinning the need of further validation studies. Although the D-RECT-German could be a helpful tool to evaluate further medical education in the German speaking area the present state of evidence highly demands further studies to examine criterias for test quality. For international comparisons of the two instruments the global score might be used with reservations, until other studies with the D-RECT have been completed. 

## 6. Acknowledgements

The authors thank all residents and attendings who participated in this study, in partcular Dr. Klarke Boor, PhD, Amsterdam, Niederlanden for her support and Professor Dr. med. Martin R. Fischer, LMU Munich, Germany for his important impetus in the practical implementation of this study. 

## Competing interests

The authors declare that they have no competing interests. 

## Supplementary Material

Represented are item and scale characteristics of the items from D-RECT (M: mean; SD: standard deviation, rit: discriminatory power according to Pearson, α=Cronbach’s α). The evaluation occurs using a five-point Likert Scale (see paragraph 2.1). #=Reference data (Boor et al., [7]); Items are ordered by the subscales / factors 1. – 11. of the original [7].

## Figures and Tables

**Table 1:**
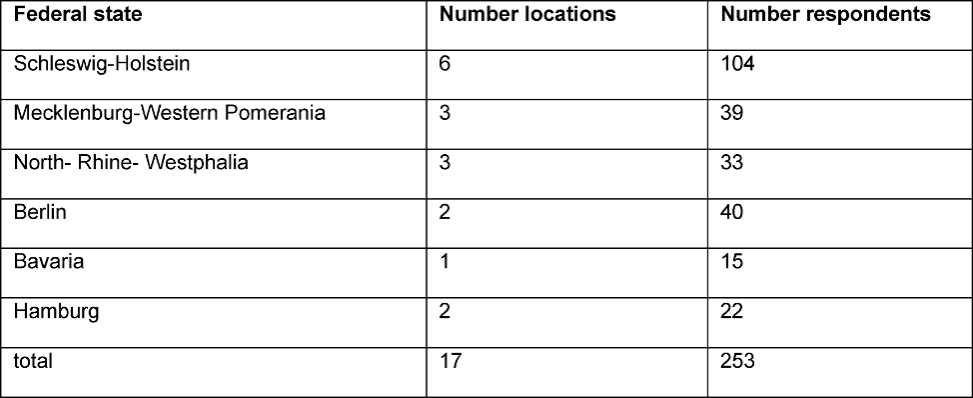
Differentiation of respondents with respect to federal states and locations (n=2: missing).

**Table 2:**
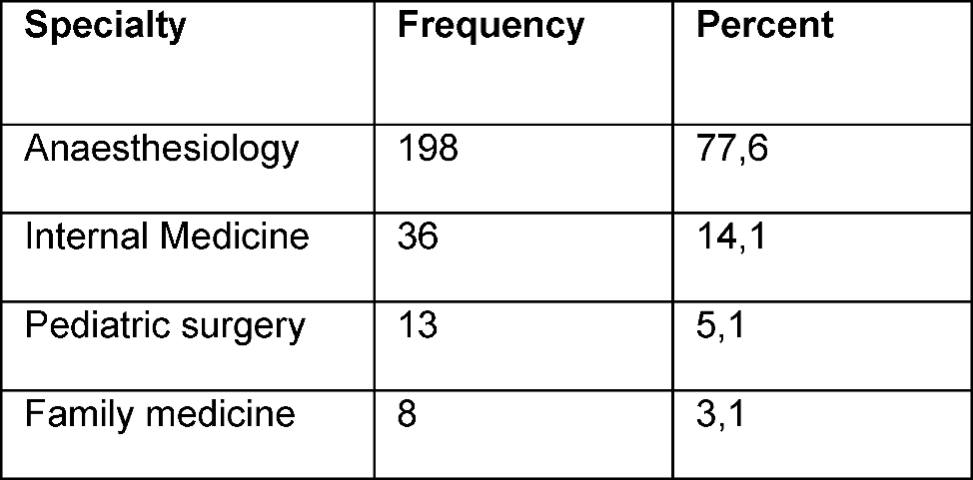
Differentiation of respondents with respect to specialties.

**Table 3:**
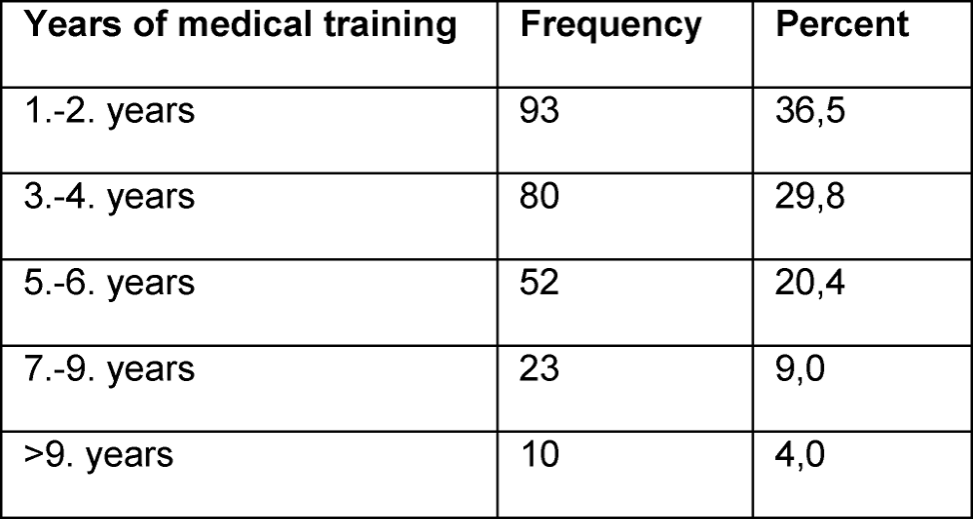
Differentiation of respondents with respect to to years of medical training.

**Table 4:**
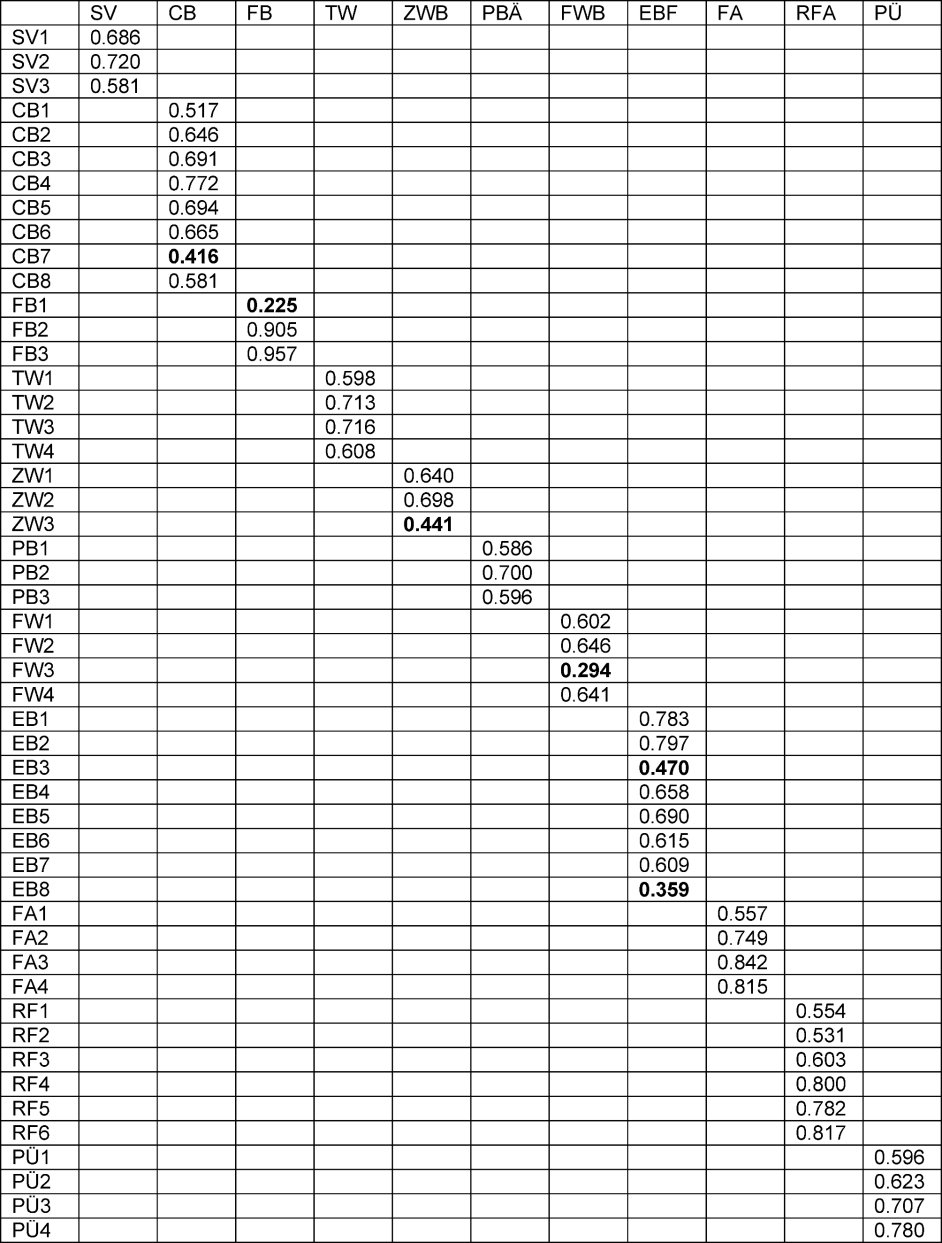
Factor loadings (standardized regression coefficients rs between items and dimensions). Abbreviations see attachment 1.

**Table 5:**
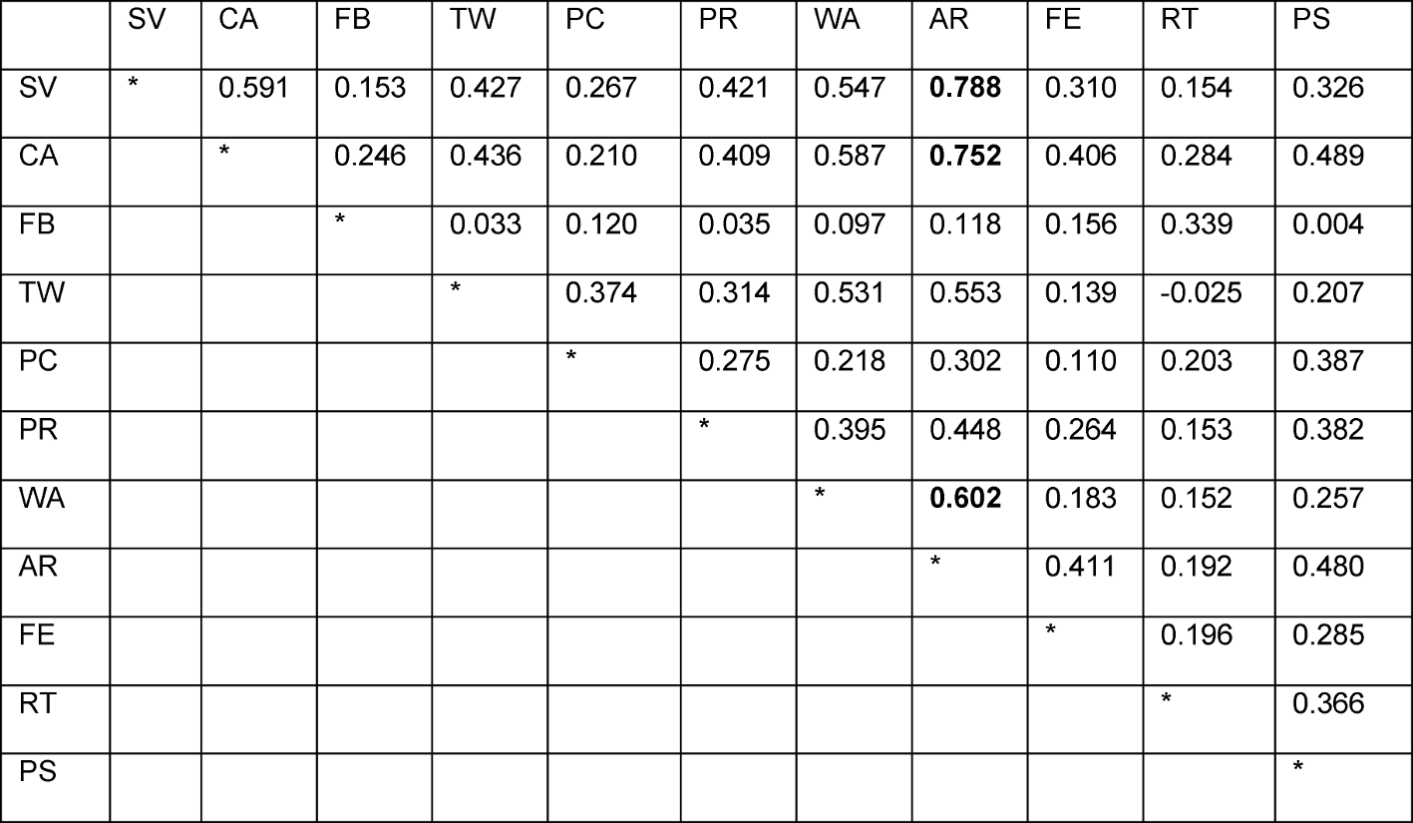
Correlations r between the subscales. Abbreviations see attachment 1.
