# Supplementary material for: The Questionnaire D-RECT German: Adaptation and testtheoretical properties of an instrument for Evaluation of the learning climate in medical specialist training
Source: GMS Z Med Ausbild. 2015 Nov 16;32(5):Doc55. doi: 10.3205/zma000997 (PMC4647162; doi:10.3205/zma000997)
Supplement: Represented are item and scale characteristics of the items from D-RECT (M: mean; SD: standard deviation, rit: discriminatory power according to Pearson, α=Cronbach’s α). The evaluation occurs using a five-point Likert Scale (see paragraph 2.1). #=Reference data (Boor et al., [7]); Items are ordered [file ZMA-32-55-s-001.pdf]

	Learning climate-Scales/ Factors	M	SD	r_{it}	α	M#	SD#	α#
	1. Supervision	3.38	0.78		.584			.64
1	SV1: The guidelines clearly outline when to request input from a supervisor.	2.66	1.17	.605		4.26	0.95	
2	SV2: The amount of supervision I receive is appropriate to my level of experience.	3.24	1.04	.596		4.09	0.91	
3	SV3: It is clear which attending supervises me.	4.31	0.87	.347		4.26	0.95	
	2. Coaching & Assessment	2.24	0.71		.833			.80
4	CA1: I am asked on a regular basis to provide a rationale for my management decisions and actions.	2.60	1.00	.465		3.88	.88	
5	CA2: My attendings coach me on how to communicate with difficult patients.	2.10	1.04	.563		3.42	1.03	
6	CA3: My attendings take the initiative to explain their actions.	2.83	0.94	.602		3.46	0.98	
7	CA4: My attendings take the initiative to evaluate my performance.	2.32	1.03	.621		3.08	1.06	
8	CA5: My attendings take the initiative to evaluate difficult situations I have been involved in.	2.21	1.00	.563		3.01	1.04	
9	CA6: My attendings evaluate whether my performance in patient care is commensurate with my level of training.	2.18	1.11	.597		3.40	1.09	
10	CA7: My attendings occasionally observe me taking a history.	1.36	0.79	.354		2.85	1.22	
11	CA8: My attendings assess not only my medical expertise but also other skills such as teamwork, organization or professional behavior.	2.33	1.33	.550		3.67	1.12	
	3. Feedback	1.52	0.53		.569			.75
12	FB1: My attendings give regular feedback on my strengths and weaknesses.	2.37	0.99	.647		3.37	1.14	
13	FB2: Observation forms (i. e. Mini-CEX) are used to structure feedback.	1.10	0.56	.248		3.81	1.19	
14	FB3: Observation forms (i. e. Mini-CEX) are used periodically to monitor my progress.	1.10	0.55	.255		3.42	1.27	
	4. Teamwork	3.41	0.74		.737			.69
15	TW1: Attendings, nursing staff, other allied health professionals and residents work together as a team.	3.68	0.81	.422		3.82	1.00	
16	TW2: Nursing staff and other allied health professionals make a positive contribution to my training.	3.60	0.93	.305		3.66	1.09	
17	TW3: Nursing staff and other allied health professionals are willing to reflect with me on the delivery of patient care.	3.22	0.94	.378		3.76	1.02	
1	TW4: Teamwork is an integral part of	3.14	1.25	.397		3.00	1.04	

8	my training.							
	5. Peer Collaboration	3.70	0.81	.	.583			.76
19	PC1: Residents work well together.	3.99	0.83	.351		4.48	.79	
20	PC2: Residents, as a group, make sure the day's work gets done.	3.68	1.19	.274		4.14	1.04	
21	PC3: Within our group of residents it is easy to find someone to cover or exchange a call.	3.42	1.24	.267		4.26	.91	
	6. Professional relations between attendings	2.64	0.91		.658			.77
22	PR1: Continuity of care is not affected by differences of opinion between attendings.	3.23	1.16	.374		3.76	1.12	
23	PR2: Differences of opinion between attendings about patient management are discussed in such a matter that is instructive to others present.	2.50	1.10	.441		3.50	1.14	
24	PR3: Differences of opinion are not such that they have a negative impact on the work climate.	2.18	1.29	.343		3.62	1.16	
	7. Work is adapted to residents' competence	3.26	0.69		.591			.66
25	WA1: The work I am doing is commensurate with my level of experience.	3.81	0.81	.383		4.12	0.84	
26	WA2: The work I am doing suits my learning objectives at this stage of my training.	3.44	0.89	.420		3.97	0.92	
27	WA3: It is possible to do follow up with patients.	3.00	1.36	.222		3.96	1.08	
28	WA4: There is enough time in the schedule for me to learn new skills.	2.79	0.99	.463		3.24	1.08	
	8. Attendings' role	3.67	0.68		.832			.85
29	AR1: My attendings take time to explain things when asked for advice.	3.71	0.88	.606		4.12	0.80	
30	AR2: 30 My attendings are happy to discuss patient care.	3.32	0.92	.671		4.16	0.82	
31	AR3: There is (are) NO attending physician(s) who have a negative impact on the educational climate.	2.49	1.45	.509		3.34	1.27	
32	AR4: My attendings treat me as an individual.	3.94	0.97	.506		3.47	1.18	
33	AR5: My attendings treat me with respect.	4.09	0.84	.541		4.46	0.87	
34	AR6: My attendings are all in their own way positive role models.	3.08	1.12	.582		3.48	1.04	
35	AR7: When I need a attending, I can always contact one.	4.42	0.80	.404		4.55	.69	
36	AR8: When I need to consult a attending, they are readily available.	4.22	0.84	.458		4.52	.72	
	9. Formal education	2.92	0.98		.829			.75
3	FE1: Residents are generally able to	3.12	1.15	.345		3.60	1.06	

7	attend scheduled educational activities.							
38	FE2: Educational activities take place as scheduled.	3.16	1.33	.320		3.89	0.98	
39	FE3: Attendings contribute actively to the delivery of high-quality formal education.	2.90	1.19	.433		3.63	1.14	
40	FE4: Formal education and training activities are appropriate to my needs.	2.49	1.14	.507		3.61	1.06	
	10. Role of the specialty tutor	1.74	1.07		.847			.78
41	RT1: The specialty tutor monitors the progress of my training.	2.30	1.47	.508		4.49	.84	
42	RT2: The specialty tutor provides guidance to other attendings when needed.	1.59	1.43	.357		3.46	1.26	
43	RT3: The specialty tutor is actively involved in improving the quality of education and training.	1.92	1.39	.442		4.12	1.06	
44	RT4: In this rotation evaluations are useful discussions about my performance.	1.59	1.45	.401		3.65	1.11	
45	RT5: My plans for the future are part of the discussion.	1.71	1.49	.366		3.61	1.16	
46	RT6: During evaluations, input from several attendings is considered.	1.34	1.33	.398		3.83	1.17	
	11. Patient sign out	4.14	0.88		.766			.75
47	PS1: When there is criticism of a management plan I have developed in consultation with my attending physician, I know the attending physician will back me up.	3.03	1.53	.510		3.74	1.13	
48	PS2: Sign out takes place in a safe climate.	3.06	1.37	.431		4.02	1.04	
49	PS3: Sign out is used as a teaching opportunity.	2.22	1.10	.445		3.96	1.06	
50	PS4: Attendings encourage residents to join in the discussion during sign out.	2.21	1.37	.521		3.69	1.15	

Table 1: Represented are item and scale characteristics of the items from D-RECT (M: mean; SD: standard deviation, r_{it}: discriminatory power according to Pearson, α=Cronbach's α). The evaluation occurs using a five-point Likert Scale (see paragraph 2.1). $^{\#}$=Reference data (Boor et al., [7]); Items are ordered by the subscales / factors 1. – 11. of the original [7].
